# Determining the Individual Theta Frequency for Associative Memory Targeted Personalized Transcranial Brain Stimulation

**DOI:** 10.3390/jpm12091367

**Published:** 2022-08-24

**Authors:** Jovana Bjekić, Dunja Paunovic, Marko Živanović, Marija Stanković, Inga Griskova-Bulanova, Saša R. Filipović

**Affiliations:** 1 Human Neuroscience Group, Institute for Medical Research, University of Belgrade, 11000 Belgrade, Serbia; 2 Institute of Psychology and Laboratory for Research of Individual Differences, Department of Psychology, Faculty of Philosophy, University of Belgrade, 11000 Belgrade, Serbia; 3 Institute of Biosciences, Life Sciences Centre, Vilnius University, LT-10322 Vilnius, Lithuania

**Keywords:** individual theta frequency (ITF), EEG, associative memory, brain stimulation, personalization

## Abstract

Non-invasive brain stimulation (NIBS) methods have gained increased interest in research and therapy of associative memory (AM) and its impairments. However, the one-size-fits-all approach yields inconsistent findings, thus putting forward the need for electroencephalography (EEG)-guided personalized frequency-modulated NIBS protocols to increase the focality and the effectiveness of the interventions. Still, extraction of individual frequency, especially in the theta band, turned out to be a challenging task. Here we present an approach to extracting the individual theta-band frequency (ITF) from EEG signals recorded during the AM task. The method showed a 93% success rate, good reliability, and the full range of variability of the extracted ITFs. This paper provides a rationale behind the adopted approach and critically evaluates it in comparison to the alternative methods that have been reported in the literature. Finally, we discuss how it could be used as an input parameter for personalized frequency-modulated NIBS approaches—transcranial alternating current stimulation (tACS) and transcranial oscillatory current stimulation (otDCS) directed at AM neuromodulation.

## 1. Introduction

Oscillatory brain activity in different frequency bands (i.e., delta (0.5–4 Hz), theta (4–8 Hz), alpha (8–12 Hz), beta (12–30 Hz), and gamma (30–80 Hz)) has been related to the different cognitive processes. The functional significance of rhythmic brain activity led to the increased interest in using non-invasive brain stimulation (NIBS) techniques for the direct modulation of these oscillations [1]. Specifically, transcranial electrical stimulation (tES) techniques, such as transcranial alternating current stimulation (tACS) and oscillatory transcranial direct current stimulation (otDCS), have received considerable attention [2,3,4]. In both techniques, weak sinusoidally modulated electric current is delivered to affect endogenous neural oscillations [5], and consequently, influence performance via network-wide recruitment [6]. The tACS/otDCS can change brain activity either by matching the intrinsic frequencies with exogenous input to increase the amplitude of the intrinsic oscillations (resonance), or by constraining/entraining brain oscillatory activity into the desired frequency by stimulating regardless of the frequency of the intrinsic oscillations [5,7].

To modulate cognitive functions, most tACS/otDCS studies used mid-band frequencies across all participants, e.g., 10 Hz for “alpha-band stimulation” (for review see [7,8]). However, this one-size-fits-all approach resulted in inconsistent findings, thus putting forward the need for personalized frequency-specific NIBS to increase its effectiveness in research and therapy (e.g., see [9,10]). This entails tuning the stimulation waveform to the endogenous network dynamics by using the individual peak frequency of the targeted oscillation [10]. However, finding the individual peak frequency can be a challenge when seeking to personalize the stimulation outside the alpha-band. In this paper we focus on determining the dominant theta-band frequency for associative memory (AM) targeted NIBS.

Theta oscillations dominate hippocampal electrical activity [11] and are considered to play a crucial role in the hippocampal–neocortical interactions [12,13], as well as in the interactions across the widespread neocortical circuits [14]. Theta oscillations have long been implicated in learning and memory [15], that is, theta activity was found to be associated with information and contextual processing [16], temporal organization for memory engrams [17,18], and associative binding [19]. Furthermore, theta activity has been observed during lower-level mnemonic processes such as encoding, recognition, and recall [20]. Recent reviews argue that theta-band oscillations are causally engaged in AM [15].

Since a prominent peak in theta activity is usually not observed in EEG frequency spectra, determining individual-specific dominant theta frequency is not a straightforward task. Probably, this is the reason that there have been only a few attempts to deliver theta frequency-personalized tES [21,22,23]. They used different methods to determine endogenous peak theta frequency or, so-called individual theta frequency (ITF). The first method relied on the most pronounced peak in the frequency power spectrum of the resting state EEG—the peak of the posterior background activity, i.e., alpha peak, or the so-called “individual alpha frequency” (IAF). The IAF is used as an anchor for determining personalized windows for other frequency bands [24]. Following this approach, Jaušovec and colleagues [21,22], applied oscillating currents at ITF for working memory neuromodulation. In their studies, ITF was determined by subtracting 5 Hz from the resting state IAF, i.e., IAF minus 5 Hz = ITF (e.g., person 1: IAF = 9.5, ITF = 4.5; person 2: IAF = 11, ITF = 6, etc.). The second approach to determining ITF is based on the cross-frequency theta-gamma coupling. It relies on the cross-frequency covariance and is built upon the evidence on the theta-gamma phase coupling in the hippocampus as a direct substrate for memory processes [13,17]. Determining ITF based on theta-gamma frequency coupling assumes finding the theta band frequency that shows the highest correlation with gamma band frequency, which is usually done through quantifying phase-amplitude coupling [25]. This approach has been adopted by different studies aiming to modulate working memory using NIBS (see [26] for review). Finally, the most straightforward approach to extracting ITF would be to find the theta band frequency with the highest power during a function-relevant task. This approach was adopted by van Driel and colleagues [23] who used the automatic peak frequency detection method to extract theta frequency with the maximal power from the EEG activity during the cognitive inhibition task.

However, none of the previous studies delivered ITF-personalized NIBS to enhance AM specifically, and there is no evidence that any of these ITF extraction approaches capture AM-relevant EEG activity. Therefore, to set the ground for future basic research and clinical trials assessing AM-targeted personalized NIBS, we present a different approach to extracting ITF and discuss how it can be applied in AM targeted tACS/otDCS studies. Here, the ITF is defined as the dominant frequency within the band (4–8 Hz) during successful AM encoding.

## 2. Materials and Methods

### 2.1. Participants

Forty-two young healthy adults (age: 22–34 years, *M* = 25.05, *SD* = 3.55; 26 females) took part in the study. Data presented here are a part of the larger project aimed at the assessment of multiple personalized oscillatory tES techniques for AM modulation (for the study protocol see [27]). All participants were right-handed (Edinburgh Handedness Inventory [28] laterality quotient > 80), with normal or corrected-to-normal vision, and they all fulfilled common tDCS inclusion/exclusion criteria, i.e., reported no history of neurological or psychiatric disorders, traumatic brain injury, or having metal implants in the head. All participants gave their written informed consent and received monetary compensation for their involvement. The study was conducted in line with the guidelines of the Declaration of Helsinki and was approved by the Institutional Ethics Committee (EO129/2020).

### 2.2. Associative Memory Task

**Figure 1:**
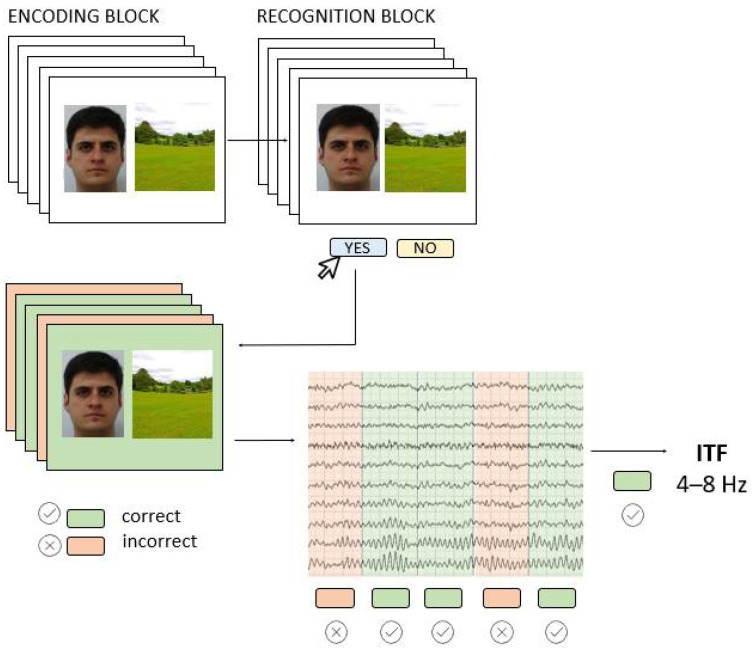
The AM task (Encoding and Recognition block) was performed while EEG was recorded; the EEG signals from the Encoding block were used to extract individual theta frequencies (ITF) for subsequently correctly recognized pairs.

To induce AM-related EEG activity, we used a visual paired associates task consisting of Encoding and associative Recognition block (Figure 1). In the Encoding block, 42 face-scene pairs were successively presented for 2000 ms, and participants were instructed to remember them as pairs. The inter-stimulus-interval (ISI) randomly varied between 1250 ms and 1750 ms, and during this time a white screen with a black fixation dot was presented. The stimuli were portrait pictures of young Caucasians of both sexes taken from FEI database [29] while scenes were publicly available pictures of all-natural scenery such as forests, seacoasts, and fields. In the Recognition block, participants saw 84 face-scene pairs, half of which were correctly paired, i.e., the same pairs they have previously seen in the Encoding block, while the other half were recombined pairs that consisted of wrongly paired faces and scenes presented in the Encoding block. The pairs were presented successively, and the participant’s task was to recognize the pair as either “old” or “recombined” by pressing one of the assigned keyboard keys. The full task code with integrated EEG triggers is made available at https://osf.io/be8df/ (accessed on 11 May 2022). The AM task was programmed and administered in OpenSesame software [30] and presented on a 23-inch monitor (0.6 m head-to-screen distance). 

### 2.3. EEG Recording

In each session, we recorded EEG first during resting state (rsEEG), 3 min eyes closed and 3 min eyes open, and then during the AM task. EEG was recorded using a light, mobile, battery-operated, hybrid tDCS-EEG Starstim^®^ device (Neuroelectrics Inc., Barcelona, Spain), which was remotely operated via Neuroelectrics^®^ Instrument Controller (NIC2) software (Neuroelectrics Inc., Barcelona, Spain). The signal was recorded with Ag/AgCl electrodes (4 mm diameter, 1 cm^2^ gel-contact area) from 20 positions (Fp1, Fp2, Fz, F3, F4, F7, F8, T7, T8, Cz C3, C4, CP5, CP6, Pz, P3, P4, PO7, PO8, and Oz according to the international 10–10 EEG positioning system). For the reference (CMS) and ground (DLR), we used either an ear-clip with a dual CMS-DLR electrode on the right earlobe or pre-gelled adhesive electrodes on the right mastoid, depending on the signal quality. The impedance was kept below 5 kΩ throughout the recording. The EEG signals were recorded with the sampling rate of 500 Hz, 0–125 Hz (DC coupled) bandwidth, and 24 bits-0.05 µV resolution. The offline signal preprocessing was performed in the EEGLAB for MATLAB [31] separately for resting state (rsEEG) and the task-EEG. The signal was high-pass filtered at 0.1 Hz and the power-line noise (50 Hz) was removed using multi-tapering and Thomas F statistics, as implemented in the CleanLine plugin for EEGLAB. The channels with substantial noise were excluded after visual inspection. The independent component analysis (ICA), using “runica” routine with default settings, was performed on the remaining channels to detect and remove eye movement artefacts. 

The EEG signal recorded during the Encoding block of the AM task was analyzed to extract ITF. The epochs were created from −1000 ms to 2500 ms in respect to the stimulus onset and baseline-corrected to the pre-stimulus period (−800 ms to 0 ms). The data were visually inspected, and bad epochs (i.e., the epochs with artifacts) were manually rejected. The epochs were labeled based on the subsequent recognition accuracy. Namely, the epochs containing encoding stimuli that were correctly identified as “old” in the recognition block were labeled as “successful encoding” and the trials that were incorrectly identified as “recombined” were labeled as “unsuccessful encoding”. 

### 2.4. Individual Theta Frequency (ITF)

Since in this study we were interested in the AM-related theta activity, the ITF was defined as the dominant theta-band frequency during successful AM encoding. Therefore, the ITF extraction was performed on “successful encoding” epochs within the time-window from 250 ms to 1250 ms after the stimulus onset covering the time-window when AM-related EEG activity has been usually recorded [32].

The signal was subjected to time-frequency transformation using complex Morlet wavelet (7 cycles) to extract frequencies from 1 to 15 Hz in 0.5 Hz resolution using MATLAB Wavelet Toolbox. To calculate the event-related spectral perturbation (ERSP) indicating event-related changes in power relative to a pre-stimulus baseline, we used the formula:

$$ERSP\left(c,f,t\right)=\frac{1}{N}\overset{N}{\underset{n}{\sum }}{\left|X\left(c,f,t,n\right)\right|}^{2}$$

where for every channel *c*, frequency *f*, and time point *t*, a measure is calculated by taking time-frequency decomposition *X* of each trial *n* [33]. The ERSP was further expressed as a ratio against the baseline (−800 ms to 0 ms) for each 0.5 Hz, starting from 2 to 15 Hz. The ERSP calculations were performed on each trial separately and then averaged to obtain an ERSP representative for the given electrode. 

**Figure 2:**
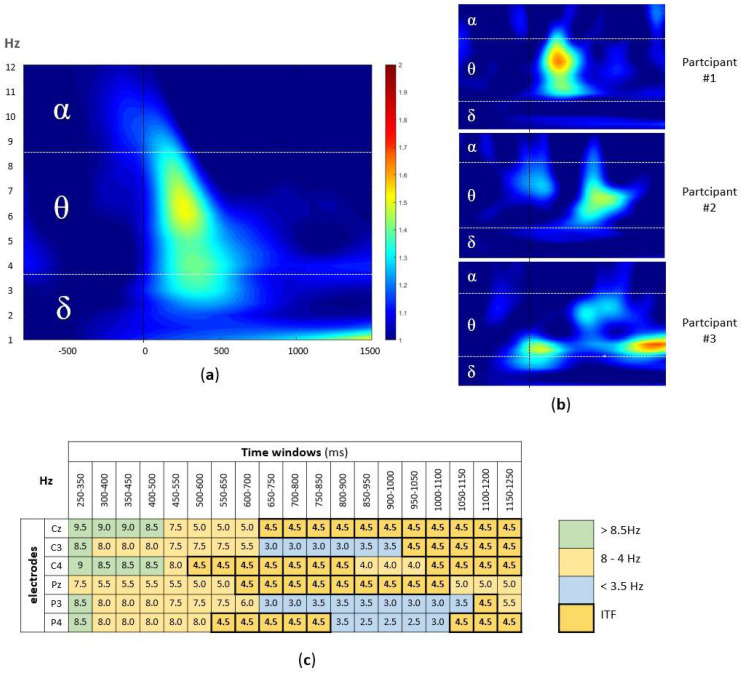
(**a**) The group-level time-frequency plot of the EEG signal during the successfully encoded pairs. The ERSP values, averaged across participants and electrodes, were plotted within the window of −800 and 1250 ms (x-axis), with stimulus onset marker (black line) and 1–12 Hz (y-axis) split into alpha, theta, and delta band. (**b**) The examples of individual time-frequency plots (three participants); the same x-axes and y-axes as well as color coding as in (**a**) were used. (**c**) The example of participant-level time x electrode matrix with Hz with highest ERSP value in each cell, marked according to the alpha (green), theta (orange), and delta (blue) band, with ITF of 4.5 Hz, i.e., modal value marked bold.

The signal from six centroparietal electrodes (Cz, C3, C4, Pz, P3, P4) was analyzed, since they cover the scalp area where the AM-related EEG activity is known to be detected [34]. At the group level, the highest activity was observed in the theta band (4–8 Hz) in the first 500 ms after the stimulus onset (Figure 2a). However, at the individual level, the differences in peak frequencies as well as latencies were observed (Figure 2b). To account for the variability in latencies, using custom-written MATLAB script, the frequency with the highest ERSP value was extracted from each of the 19 overlapping time windows (100 ms window width, in 50 ms steps; from 250 ms to 1250 ms, i.e., 250–350 ms, 300–400 ms, 350–450 ms, …, 1150–1250 ms) at each of six centroparietal electrodes (Cz, C3, C4, Pz, P3, and P4).

This resulted in the participant-level time x electrode matrix (114 cells per participant), with the frequency with the highest ERSP in each cell (Figure 2c). Finally, to extract the dominant theta band frequency, we calculated the mode (i.e., the most frequently occurring value) for the frequencies between 4–8 Hz (in 0.5 Hz steps) in the time x electrode matrix for each participant.

### 2.5. The Control Procedure 

To control for potential artifact of the ITF extraction method, we used the “eyes open” part of the rsEEG. Namely, rsEEG was pre-processed, segmented into successive 3500 ms epochs (first 1000 ms serving as baseline), and the same analysis as described above was conducted. 

### 2.6. Statistical Analysis 

The statistical analyses were performed using the Analyze Data module in Microsoft Excel and JASP (JASP, 2022). As the measure of AM performance, we calculated the number and the percentage of correctly identified targets and correctly rejected recombined pairs as well as the overall success rate. For AM measures and AM-related ITF, descriptive statistics, such as mean, standard deviation, median, range, etc., were calculated. In addition, the relative share of theta-band frequencies in time x electrode matrix was calculated (i.e., the percentage of cells with the highest ERSP in the theta range in comparison to the neighboring frequency-bands, i.e., delta and alpha), as well as the relative share of the ITF in cells with theta-band frequencies (i.e., the percentage of cells with the ITF out of all cells with frequencies within theta band). The latter was interpreted as the participant-level reliability of the extracted ITF. For rsEEG data, the relative share (percentage) of alpha, theta, and delta band frequencies in time x electrode matrix, as well as the share of theta-band frequencies in comparison to the neighboring frequency-bands, were calculated.

## 3. Results

**Figure 3:**
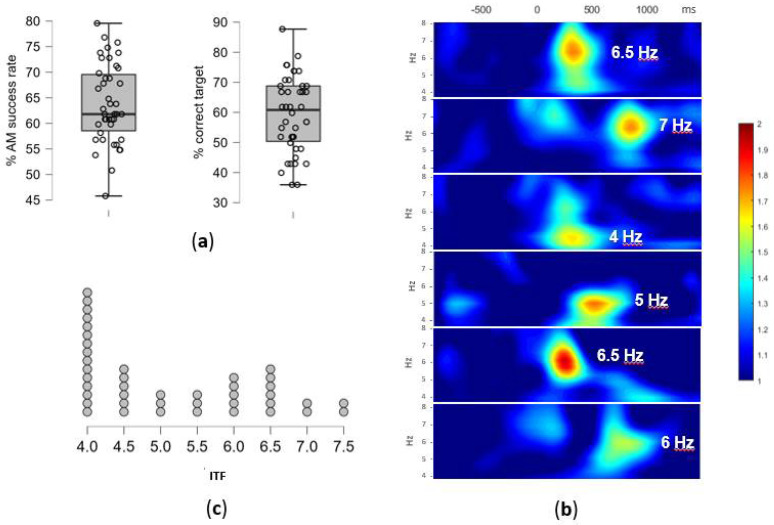
(**a**) The AM task performance: the overall success rate in % and the % of correctly identified targets, i.e., successfully encoded pairs. Individual participants overlay the box-plot marking median and interquartile range (**b**) The individual differences in theta activity during the AM encoding: examples of time-frequency plots of the EEG signal during the successfully encoded pairs for six participants; the ERSP values, averaged across 9 centro-parietal electrodes, are plotted within the window of −800 and 1250 ms (x-axis) and 4–8 Hz (y-axis), with the extracted ITF for each participant marked; (**c**) The individual theta frequency (ITF) distribution: the number of participants and their extracted ITF values in Hz (x-axis).

The overall success rate in AM task was on average 64.0% (*SD* = 7.70), with an average of 68.5% (*SD* = 13.19) for correctly rejected recombined pairs and 59.5% (*SD* = 12.72) for correctly identified targets (Figure 3a). As encoding epochs were selected based on subsequent associative recognition (“successfully encoded”), this resulted in 25 epochs on average (out of 42 in total) to be used for ITF extraction. However, due to substantial variability in memory performance, the number of epochs on the individual level was between 15 and 37. After the removal of bad epochs from the EEG signal, between 14 and 37 (*M* = 23.57, *SD* = 5.71) epochs remained for the analysis.

The individual differences in the peak theta frequency as well as the time-range of enhanced theta-band activity occurrence during successful AM encoding are presented in selected time x frequency plots on Figure 3b. The time x electrode matrix for each participant showed high inter-individual variability in the theta-activity dominance (the number of cells with highest ERSP in the theta range in comparison to the neighboring frequency-bands i.e., delta and alpha). The ERSP peaks in the theta-band frequencies have been observed in 51.5% of cells on average (*SD* = 24.1%), with the high inter-individual variability (range: 7.9–92.1%). The remaining cells had peaks in delta (38.6%, *SD* = 28.7, range: 0–85.1 %) and alpha bands (9.9%, *SD* = 15.3, range: 0–69.3%). 

The ITF, defined as the modal theta-band frequency, was successfully extracted for 39 participants (93%), while for the remaining three additional steps in the analyses had to be performed. Namely, one participant had double mode distribution of frequencies (7.5 Hz between 650–950 ms and 5.5 Hz between 950–1200 ms on all electrodes), and two participants did not have a prominent theta activity in the defined time x electrode matrix, thus EEG signal from additional electrodes (PO7 and PO8) was analyzed to extract ITF. Eventually, ITF ranged between 4 Hz and 7.5 Hz (*M* = 5.16, *SD* = 1.16), with the distribution presented in Figure 3c.

However, it is important to note that there was significant variability between participants in homogeneity of peak theta-frequencies, i.e., in occurrence of ITF within theta-band frequencies during the successful AM encoding. Namely, among all peak frequencies in the theta band, identified by the previously described method, the ones singled-out as the ITFs were observed in 26% to 100% of cells, depending on the participant (53% on average, *SD* = 20%). Thus, based on the incidence, the ITFs could be classified as: singular ITF [>80% (*n* = 5)], highly reliable ITF [51–80% (*n* = 13)], reliable ITF [31–50% (*n* = 18)], unreliable ITF [15–30% (*n* = 8)], and no ITF [<15%, i.e., below the chance level (*n* = 0)].

In the control condition, i.e., eyes open rsEEG, the method extracted theta-band peaks in only 33.8% of cells (*SD* = 27.2, range 0–79.8%), which was significantly less than in AM condition (*p* < 0.05). Most of the ERSP peaks in rsEEG were observed in alpha-band (41.8% of cells on average, *SD* = 29.6, range: 0–100%), while the relative share of delta-band peaks was 24.4% (*SD* = 27.6, range: 0–94.7%)

## 4. Discussion

The study shows the feasibility of extracting ITF from AM-relevant neurophysiological activity that can be used as an input parameter for frequency-personalized tACS/otDCS aimed at memory neuromodulation through entrainment of the cortico-hippocampal circuits. Previous NIBS studies that used theta band stimulation typically applied a single stimulation frequency within theta band which was the same for all participants: either 4 Hz [35,36], 5 Hz [37,38,39], or 6 Hz [40,41,42,43,44,45,46,47,48]. However, there is evidence suggesting that the choice of stimulation frequency may be a relevant factor that can affect the NIBS effects—for example, two studies that contrasted effects of low (4 Hz) and high (7 Hz) theta tACS found differential effects on memory [49,50]. Since the tACS/otDCS effects are expected to be the most prominent when the stimulation frequency is close to a person’s endogenous peak or dominant frequency [37,51], finding the function-relevant ITF is an important step towards increasing the NIBS effectiveness through delivering personalized frequency-modulated stimulation. To the best of our knowledge, this is the first successful attempt to extract ITF from the AM-task-related EEG.

We conceptualized ITF as the dominant theta-band frequency during successful AM encoding. Hence, to capture individual differences in the AM encoding time-frequency spectra, a new approach for ITF extraction needed to be developed. Similar to van Driel and colleagues [23], we opted for an approach that is based on the differences in power between theta-band frequencies. However, since automatic peak detection cannot ensure that identified ITF has sufficiently higher power than other frequencies, and since the latencies of the strongest theta synchronization differed between the participants, we decided to extract peak frequencies across multiple time windows and multiple electrodes. This does not change the basic assumptions of a power-based approach but provides a more reliable ITF estimate due to the increased number of observations. The ITF extraction based on the modal theta-band frequency across multiple time-windows and electrodes resulted in 93% of successfully identified dominant frequencies, while the remaining cases could be resolved by additional analysis of neighboring electrodes. Still, it is important to note that even with the relatively large number of observations (more than 100), it can be difficult to identify a single modal value. This opens the question if the ITF-based personalization of the tACS/otDCS should be understood as a continuum, that is, whether we need to assess and report the level of NIBS personalization based on the reliability of ITF. The ITF extraction approach proposed here allows for that possibility by a simple calculation of the proportion of cells in time x electrode matrix in which ITF value appears for each participant. Namely, if we treat value (i.e., the frequency with the highest ERSP) within the time x electrode matrix as a repeated measure of the same underlying process responsible for successful encoding, the proportion of occurrence of each value can be interpreted as their reliability in repeated measurements. For example, if an ITF occurs in 70% of the “measurements” across time and electrodes, a given value can be considered highly reliable and therefore inherent for successful encoding of a given individual. Consequently, when used as an input parameter for tACS/otDCS, the protocol can be considered as highly personalized. Conversely, if a participant’s ITF occurs in fewer than 30% of the total measurements, i.e., has the reliability of <0.30, a given value cannot be regarded as reliable and thus ITF-based tACS/otDCS protocol could not be labeled as well-personalized.

Since ITF was extracted from the task-related EEG signal, several aspects of the AM task itself need to be emphasized. First, the AM was operationalized by paired-associates task typically used in AM-NIBS studies [52,53,54,55,56,57,58]. The task was designed to be of the optimal difficulty for young healthy volunteers, and as such, it cannot be used in older adults or clinical populations without adjustments or prior testing. Furthermore, the individual differences in AM performance resulted in a variable number of epochs to be analyzed. The number of epochs may be smaller than what is recommended for a reliable EEG analysis. However, this issue cannot be addressed simply by increasing the number of encoding trials as that would increase the difficulty of the task and probably result in higher chance-based responses in the recognition phase. Second, we opted for analyzing the EEG signal from encoding rather than recognition blocks, based on the assumption that theta-rhythm underlies the process of binding, that is, creating new associations in memory [59,60,61], and that the encoding EEG is free of other processes involved in the task-based-recognition (e.g., decision making, motor-control/action). Finally, the stimuli were selected to be as ecologically valid as possible (human faces and landscapes); however, further studies are needed to show that the same ITFs could be extracted from the same-structure task using different types of stimuli.

Our data suggest that, on the group level, theta-band activity underlines associative encoding. What is more, the developed analytical method produces significantly less theta-peaks in the control condition (i.e., rsEEG) providing further confirmation that the extracted ITFs reflect encoding processes. However, due to the limited number of unsuccessful trials in the AM task, we cannot provide empirical evidence that the extracted ITFs are specific for successful encoding and not encoding in general. To answer this question, a specifically designed study on either lower performing samples or using a more difficult AM task is needed. Yet, it is important to note that the activity during successful encoding was not limited to theta-band. Although there is ample evidence that theta activity underlines memory, other, especially slower, oscillations might play a relevant role too. This is an issue that deserves more attention in future studies. 

The critical aspect that needs to be properly determined in future studies is the test-retest reliability of the extracted ITFs since it will determine the future use of the ITF in NIBS personalization. Namely, if ITF is found to have good reproducibility across EEG recorded in separate days, the parameter can be considered a trait-like feature and once determined can be used to personalize NIBS in subsequent sessions. However, if the test-retest reliability proves to be low, it would suggest state-dependent qualities of ITF and would call for continuous adjustment of stimulation frequency in a closed-loop manner. 

Despite these constraints, it could be argued that the proposed ITF extraction method has advantages over the other available approaches reported in literature such as analyzing individual differences in the resting state EEG [21] or the theta-gamma cross-frequency coupling ITF [62]. Namely, even though ITF defined as IAF-5 Hz ensures that the most prominent peak is used as an anchor [24], it also assumes universal equidistance between IAF and ITF across all participants, which may not be the case. More importantly, it could be argued that ITF for memory should not be directly extrapolated from the resting state EEG due to the individual differences in neural oscillators engaged at rest or during task performance. On the other hand, the cross-frequency coupling method has a stronger theoretical background as there is evidence that theta-gamma coupling in the hippocampus underlies memory processes [13,17]. However, this theta-gamma coupling-based ITF has not been evaluated in AM studies. Moreover, the phase-amplitude coupling is only one out of many possible implementations of cross-frequency coupling in neuromodulation. Namely, from the theoretical perspective in addition to the phase-amplitude coupling, the cross-frequency coordination can also be power-to-power, phase-to-phase (or phase-locking), and phase-to-frequency [63], and the recent review concludes that the relationship between different coupling phenomena and memory is still understudied [26]. Thus, although in principle this approach seems promising for the development of different long-range network multichannel tES protocols and assessing their effects (see e.g., [41]), it should be used with caution for the extraction of the ITF to serve as an input parameter for personalized NIBS protocol, as the role of coupling phenomena in AM is yet to be understood. 

Finally, the important advantage of the presented approach is the possibility of quantifying the participant-level reliability of the ITF and, consequently, the level of personalization achieved by the frequency-tuning tACS/otDCS. Adding a dimension of the quality or degree of personalization to the usual contrast personalized vs. non-personalized NIBS might help provide better understanding of uncertain tACS/otDCS effects. Namely, it is likely that the variability in responsiveness to tACS/otDCS could at least partially be explained by the level of frequency-adjustment we are able to achieve for each participant. Therefore, we argue that any method which is used to determine ITF for NIBS needs to allow for assessing and quantifying the reliability/precision of the extracted parameter on a person-by-person basis. 

## Data Availability

The datasets generated for this study are available upon a reasonable request.
